# Machine learning did not beat logistic regression in time series prediction for severe asthma exacerbations

**DOI:** 10.1038/s41598-022-24909-9

**Published:** 2022-11-27

**Authors:** Anne A. H. de Hond, Ilse M. J. Kant, Persijn J. Honkoop, Andrew D. Smith, Ewout W. Steyerberg, Jacob K. Sont

**Affiliations:** 1 Department of Information Technology and Digital Innovation, Leiden University Medical Centre, Albinusdreef 2, 2300 RC Leiden, The Netherlands; 2 Clinical AI Implementation and Research Lab, Leiden University Medical Centre, Albinusdreef 2, 2300 RC Leiden, The Netherlands; 3 Department of Biomedical Data Sciences, Leiden University Medical Centre, Albinusdreef 2, 2300 RC Leiden, the Netherlands; 4 Department of Respiratory Medicine, University Hospital Wishaw, 50 Netherton Street, Wishaw, ML2 0DP UK

**Keywords:** Machine learning, Predictive medicine, Statistical methods, Epidemiology

## Abstract

Early detection of severe asthma exacerbations through home monitoring data in patients with stable mild-to-moderate chronic asthma could help to timely adjust medication. We evaluated the potential of machine learning methods compared to a clinical rule and logistic regression to predict severe exacerbations. We used daily home monitoring data from two studies in asthma patients (development: n = 165 and validation: n = 101 patients). Two ML models (XGBoost, one class SVM) and a logistic regression model provided predictions based on peak expiratory flow and asthma symptoms. These models were compared with an asthma action plan rule. Severe exacerbations occurred in 0.2% of all daily measurements in the development (154/92,787 days) and validation cohorts (94/40,185 days). The AUC of the best performing XGBoost was 0.85 (0.82–0.87) and 0.88 (0.86–0.90) for logistic regression in the validation cohort. The XGBoost model provided overly extreme risk estimates, whereas the logistic regression underestimated predicted risks. Sensitivity and specificity were better overall for XGBoost and logistic regression compared to one class SVM and the clinical rule. We conclude that ML models did not beat logistic regression in predicting short-term severe asthma exacerbations based on home monitoring data. Clinical application remains challenging in settings with low event incidence and high false alarm rates with high sensitivity.

## Introduction

The collection of home monitoring data via mobile applications, online surveys and wearables is becoming increasingly popular to remotely monitor patients. Monitoring has the potential to aid in detecting clinical deterioration earlier, which is associated with better clinical outcomes^1^. For many applications, simple clinical rules have been developed to predict short-term events such as severe clinical deterioration^2–5^.

The advent of machine learning (ML) means we can develop highly flexible models with the ability to automatically learn from data, capture complex patterns, and incorporate time-series trends. ML models might overtake some of the moderately effective clinical rules^2–5^. ML has shown great results in application areas such as image recognition^6–8^. Its utility for home monitoring time-series data remains to be determined. Home monitoring time series data present a distinctive set of challenges for the application of ML predictive algorithms. A large effective sample size is important^9,10^, which is challenging with a low incidence of the outcome of interest. For example, severe asthma exacerbations occur in less than 0.5% of days. All the other days are normal asthma control days^9,11^. Moreover, fair external validation of ML predictive algorithms on a truly independent data is rare, commonly leading to an overoptimistic impression of predictive performance^12,13^. Due to these challenges, only few models have been developed for home monitoring data^14^, and even fewer have been externally validated.

We aim to develop and validate prediction models for short-term prediction of severe asthma exacerbations in patients with stable mild-to-moderate chronic asthma based on home monitoring data. We compare the performance of two machine learning algorithms, a statistical model, and a simple asthma action plan rule^5^.

## Results

**Figure 1:**
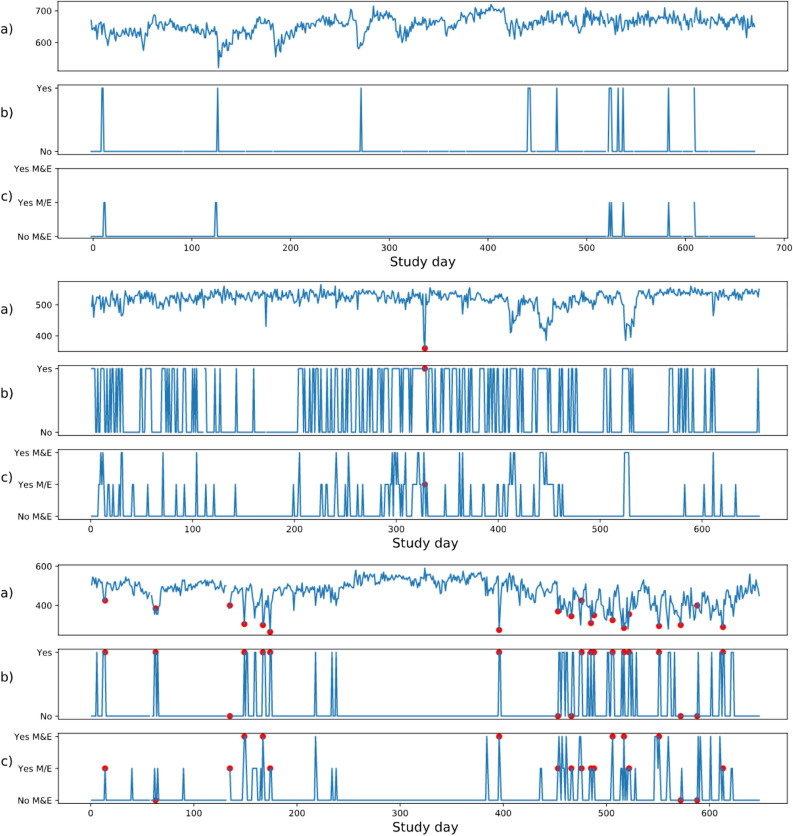
Time series for patients with no, one and many exacerbations. (**a**) Peak expiratory flow, (**b**) nocturnal awakening (yes/no), and (**c**) use of $\upbeta$2 reliever (No M&E = No Morning & Evening, Yes M/E = Yes morning or evening, Yes M&E = Yes morning and evening) over time for three patients with no, one and many exacerbations respectively. The case of no exacerbations (top figure) is most prevalent in the data. Exacerbations are marked with red dots.

The development and validation cohorts consisted of 165 and 101 asthma patients respectively (Table 1). Patients were followed for a median period of 610 days in the development and 417 days in the validation cohort. Among the development data patients, 49 had one or more exacerbations (30%). This amounted to a total of 154 exacerbations across all patients (0.2% of total 92,787 daily measurements). For the validation data this was 38 patients (38%) and a total of 94 exacerbations (also 0.2% of total 40,185 daily measurements). The percentage of missing daily measurements was below 1% for the development and below 5% for the validation cohort for all candidate predictors (Table 1). Figure 1 provides an illustration of the time series for PEF, nocturnal awakening, and use of $\upbeta$2-reliever for three representative patients with various degrees of asthma exacerbations.

**Table 1:** Descriptive statistics of the development and validation cohorts.

	Development cohort	Validation cohort
**Demographics**		
Patient, N	165	101
Total daily measurements, N	92,787	40,185
Observational period, median (25–75)	610 (580–640)	417 (376–473)
Age, median (25–75)	38 (28–47)	46.5 (34–56)
Sex (female), N (%)	92 (56%)	62 (61%)
**Predictors**		
Peak expiratory flow, mean (std)	438 (98)	404 (104)
Missing (%)	477 (0.5%)	1171 (2.9%)
Peak expiratory flow personal best^a^, mean (std)	467 (100)	437 (103)
Nocturnal awakening, mean % per patient	6.3%	4.7%
Missing (%)	876 (0.9%)	1665 (4.1%)
Use of $\upbeta$2 reliever, mean % per patient	7.2%	8.9%
Missing (%)	302 (0.3%)	1188 (3.0%)
**Outcome**		
Exacerbations per patient, N (%)		
0 exacerbations	116 (70%)	63 (62%)
1 exacerbation	25 (15%)	20 (20%)
2 or more exacerbations	24 (15%)	18 (18%)
Total exacerbations, N (%)	154 (0.2%)	94 (0.2%)

Statistics were calculated for each individual patient over their respective observational periods. Then these statistics were pooled across patients.

^a^No % missing is reported for maximum peak expiratory flow as this is a summary statistic calculated per patient over a run-in period of 4 weeks.

**Figure 2:**
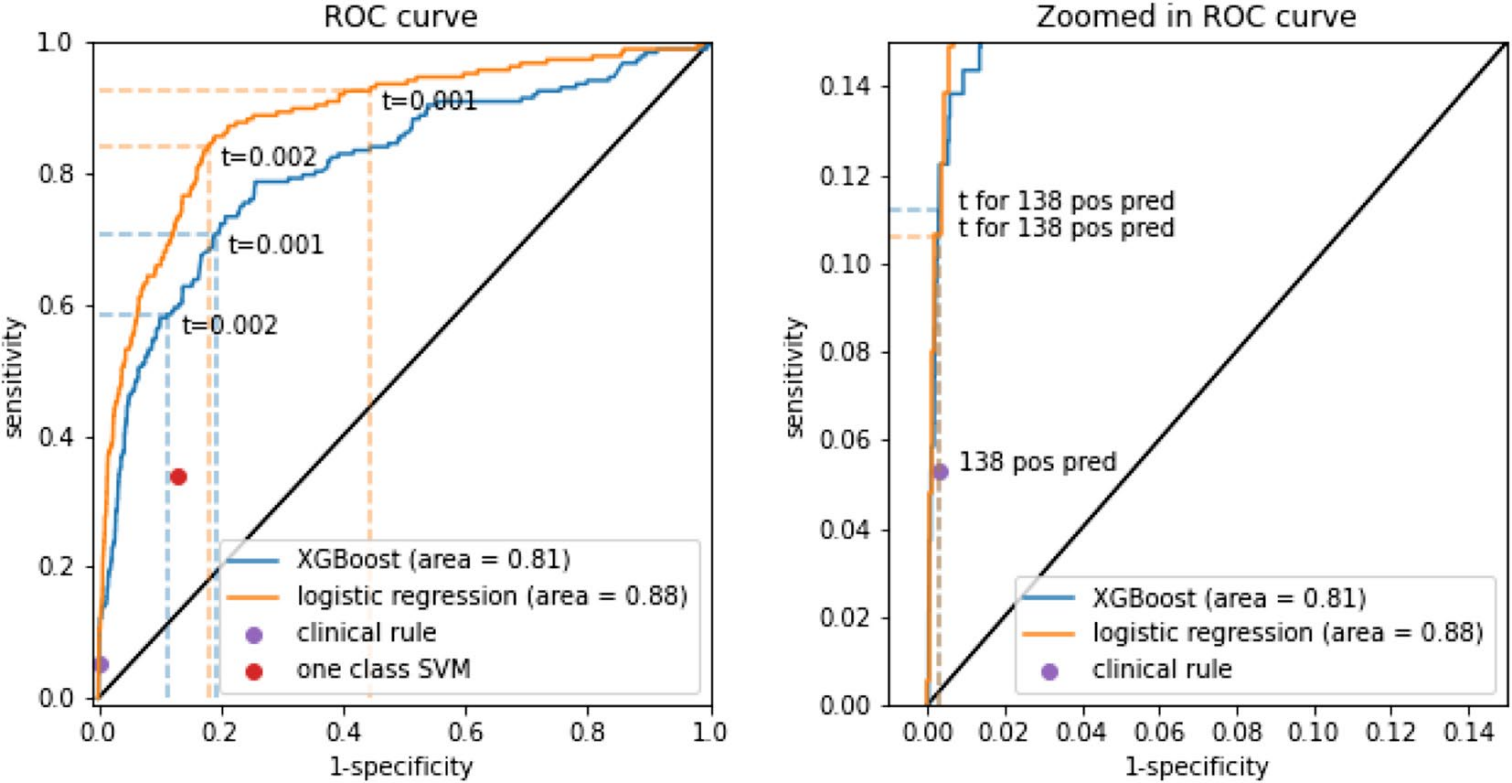
ROC-curve for predictions from XGBoost and the logistic regression model. The sensitivity and specificity of the one class SVM and clinical prediction rule are also plotted on the left curve. On the left the points corresponding to the 0.001 (‘t = 0.001’) and 0.002 (‘t = 0.002’) probability thresholds are plotted for the XGBoost and logistic regression model. On the right the points corresponding to the thresholds resulting in 138 positive predictions (‘t for 138 pos pred’, equaling the clinical rule positive predictions) are plotted for the XGBoost and logistic regression model.

XGBoost included PEF, nocturnal awakening, and use of $\upbeta$2-reliever and their corresponding statistics as predictors with first differences and first lags. At validation, the algorithm obtained an AUC of 0.81 (95% CI 0.78–0.84, Table 2, Fig. 2). The logistic regression model had a higher validated AUC of 0.88 (95% CI 0.86–0.90, p = 0.00, DeLong test). The probability distributions of the two models were heavily skewed (additional Fig. A1). Poor calibration with too extreme risk estimates was noted for the XGBoost model (calibration slope 0.56, 95% CI 0.50–0.61, Table 2, additional Fig. A2). It also underestimated the risks (calibration intercept 0.32 (95% CI 0.15–0.48). Near perfect calibration was found for the logistic regression model (slope 1.02, 95% CI 0.93–1.10, Table 2, additional Fig. A2), with some underestimation of the risk of exacerbations (intercept 0.75, 95% CI 0.60–0.90).

**Table 2:** Discrimination and calibration for predicting exacerbation within 2 days (validation cohort).

	AUC	Calibration intercept	Calibration slope
XGBoost	0.81 (0.78, 0.84)	0.32 (0.15, 0.48)	0.56 (0.5, 0.61)
Logistic regression	0.88 (0.86, 0.90)	0.75 (0.6, 0.90)	1.02 (0.93, 1.10)

*XGBoost* gradient boosted decision trees, *AUC* area under the receiver operating characteristics curve.

For the 0.2% threshold, the XGBoost model obtained a sensitivity of 0.59, a specificity of 0.89, a positive predictive value (PPV) of 0.02, and a negative predictive value (NPV) of 1 (Table 3). For the logistic regression model, this was 0.84, 0.82, 0.02, and 1 respectively.

**Table 3:** Threshold specific performance metrics for predicting exacerbation within 2 days (validation cohort).

Probability threshold	Model	Sensitivity	Specificity	PPV	NPV
0.001	XGBoost	0.71 (133/188)	0.81 (32,178/39,904)	0.02 (133/7859)	1.0 (32,178/32,233)
	Logistic regression	0.93 (174/188)	0.56 (22,227/39,904)	0.01 (174/17,851)	1.0 (22,227/22,241)
0.002	XGBoost	0.59 (110/188)	0.89 (35,326/39,904)	0.02 (110/4688)	1.0 (35,326/35,404)
	Logistic regression	0.84 (158/188)	0.82 (32,720/39,904)	0.02 (158/7342)	1.0 (32,720/32,750)
Resulting in 5217 positive predictions^b^	One class SVM	0.34 (64/188)	0.87 (34,751/39,904)	0.01 (64/5217)	1.0 (34,751/34,875)
	XGBoost	0.6 (112/188)	0.87 (34,800/39,904)	0.02 (112/5216)	1.0 (34,800/34,876)
	Logistic regression	0.73 (137/188)	0.87 (34,823/39,904)	0.03 (137/5218)	1.0 (34,823/34,874)
Resulting in 138 positive predictions^b^	Clinical rule^a^	0.05 (10/188)	1.0 (39,776/39,904)	0.07 (10/138)	1.0 (39,776/39,954)
	XGBoost	0.11 (21/188)	1.0 (39,787/39,904)	0.15 (21/138)	1.0 (39,787/39,954)
	Logistic regression	0.11 (20/188)	1.0 (39,787/39,904)	0.15 (20/137)	1.0 (39,787/39,955)

*SVM* support vector machine, *XGBoost* gradient boosted decision trees, *ppv* positive predictive value, *NPV* negative predictive value.

^a^Peak Expiratory Flow < 60% personal best.

^b^This threshold is set so that the XGBoost and logistic regression models produce the same number of positive predictions as the one class SVM or clinical rule.

The one class SVM obtained a sensitivity of 0.34, specificity of 0.87, PPV of 0.01 and NPV of 1 (Table 3). At the probability thresholds leading to the same number of positive predictions as produced by the one class SVM (5217 positive predictions), the XGBoost and logistic regression models had a higher sensitivity and PPV, and an equal specificity and NPV. The clinical prediction rule had a sensitivity of 0.05, specificity of 1, PPV of 0.07 and NPV of 1 (Table 3). With 138 positive predictions as for the clinical rule, the XGBoost and logistic regression models again had a higher sensitivity and PPV, and equal specificity and NPV.

Similar results were found for the prediction of exacerbations within 4 and 8 days as the 2-days models (additional Tables A2–A5). The AUC of the XGBoost model increased for the 5-lag model (0.85, 95% CI 0.82–0.87, additional Table A6). No such improvement for a higher number of lags was found for the logistic regression model (based on AUC, additional Table A6). The one class SVM model showed a higher sensitivity, but lower specificity for the 2-lag and 3-lag models, and a sensitivity of (almost) 1 and specificity of almost 0 for the 4-lag and 5-lag models (additional Table A7). The differences between the AUCs of the best performing logistic regression model with one lag and XGBoost model with five lags were still significant (p = 0.02, DeLong test).

## Discussion

In this study, we aimed to assess the performance of ML techniques and classic models for short-term prediction of severe asthma exacerbations based on home monitoring data. ML and logistic regression both reached higher discriminative performance than a previously proposed simple clinical rule. Logistic regression provided slightly better discriminative performance than the XGBoost algorithm. However, logistic regression still produced many false positives at high levels of sensitivity.

Our finding that ML models do not outperform classical prediction methods is in line with other recent studies^14–17^. This finding may be explained by the (lack of) complexity of the data that was studied. An advantage of ML techniques is the natural flexibility they offer to model complex (e.g. highly nonlinear) relationships, versus logistic regression techniques that have the advantage of being easily interpretable. Our findings illustrate that the flexibility provided by ML models may not always be needed to arrive at the best performing prediction model for medical data. The benefits of ML methods may differ between settings and should be further investigated.

Second, we found a substantial number of false positive predictions at high levels of sensitivity. The false positive rate (reflected by the low PPV) can be linked directly to the low incidence rate. Similar results can be found in the literature^2,18–21^. The potential implications of the high false positive rate are alarm fatigue, loss of model acceptance and trust, and ultimately disuse of the prediction model^22^. Improvement in discriminative ability may be achieved by reducing the noise in the exacerbation event at the time of data collection. For example, the recording of severe exacerbations in our dataset might have been incomplete or there might have been a delay between the recording of the exacerbations and their true onset. Moreover, better predicting variables of exacerbations may be needed, which need evaluation in large data sets.

Another insight based on our findings is that the interpretability of a prediction algorithm does not always have to come at the cost of model performance. An argument in favor of black-box ML and its broader field of artificial intelligence (AI) techniques is their potentially superior predictive performance. For this superior performance, it is deemed acceptable to not exactly know how a prediction is made: the accuracy-interpretability trade-off^23,24^. Our findings form a counterexample by showing that inherently interpretable techniques such as logistic regression may outperform ML for certain application types and clinical settings. Interpretability is especially relevant for clinical settings, as physicians often prefer interpretable models to assist in clinical decision making.

Strengths of our study include that we performed a comparison of ML models with a statistical model and a clinical prediction rule, which to our knowledge has not, or only partly been performed for this type of home monitoring data^14^. Our findings therefore contribute to answering the question when and how to apply ML methods safely and effectively, thereby putting ML in perspective. Moreover, the data used in this study contained few missing values, possibly due to the trial setting. The quality of the data was therefore high.

The current investigation also had limitations. First, by opting to predict exacerbation in the short-term (exacerbation within 2 days), the exacerbation window became small. Such a small window was chosen to keep the predictions clinically meaningful and relevant. This resulted in a very low incidence rate. We performed a sensitivity analysis in which we expanded the window to four and 8 days without noticeable differences in model performance. We therefore recommend investigating the best way to operationalize and capture the clinical definition of a severe asthma exacerbation in home monitoring data. Second, the low event rate may have caused the (best performing) logistic regression model to consistently underestimate the predicted risks^25^. Low event rates are common for the home monitoring setting. We therefore advise future researchers to investigate techniques that address any associated calibration issues. Poor calibration forms an obstacle for the implementation of any algorithm in clinical practice, since reliability of the predicted probabilities is required to be clinically meaningful^26^. Lastly, home monitoring patients based on daily diary entries can be perceived as old fashioned. Clinicians nowadays will often opt for digital telemonitoring approaches. Yet, the monitored parameters have remained largely the same across different registration modes (on paper or digitally)^18,27–29^. This implies that the registration method is unlikely to affect our conclusions.

## Conclusion

ML models may not outperform classical regression prediction model in predicting short-term asthma exacerbations based on home monitoring data. A simple regression model outperforms a simple rule. Clinical application may be challenging, due to the high false alarm rate associated with the low probability thresholds required for high sensitivity.

## Methods

### Development and validation cohorts

We analyzed two previous studies which had as the primary aim to study adjustments in asthma treatment^30,31^. The development cohort was a randomized controlled trial comparing different inhaler medications with follow up of approximately 84 weeks^31^. The validation cohort was a single-blind placebo-controlled trial examining alternative treatment pathways with follow up of approximately 60 weeks^32^. All patients had stable mild-to-moderate chronic asthma. Both studies were conducted in an asthma clinic in New Zealand on patients referred by their general practitioners. For both studies, patients recorded their peak expiratory flow and use of $\upbeta$2-reliever (yes/no) in the morning and evening of every trial day in diaries. Nocturnal awakening (yes/no) was recorded in the morning (see below).

### Outcome

The outcome variable was measured daily and was defined as the occurrence of a severe asthma exacerbation within 2 days (the day of the measurement or the following day). Table 4 provides a visualization of this 2-day window outcome. Severe asthma exacerbations were defined as the need for a course of oral corticosteroids (prednisone) for a minimum of 3 days, as documented in medical records^30,31^.

**Table 4:**
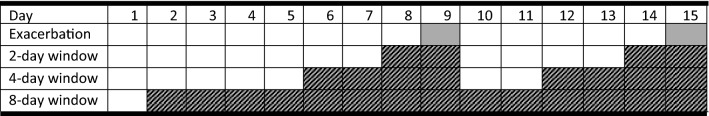
Definition of the outcome variable.

This is a hypothetical example of the definition of the outcome variable over 15 days of measurement. The patient experiences an exacerbation at day 9 and day 15. The outcome variable corresponding to a severe asthma exacerbation within 2 days is displayed on the 2-day window row. For example, at day 8 an exacerbation will occur within 2 days—it occurs the next day—and day 8 is therefore part of the 2-day window outcome. Similarly, the outcome variable definitions corresponding to exacerbations within 4 and 8 days are displayed on the 4- and 8-day window rows.

### Predictors

All predictors were measured or calculated daily. Nocturnal awakening (yes/no), the average of morning and evening peak expiratory flow (PEF, measured in liters per minute) and the use of $\upbeta$2-reliever in morning and evening (used in both morning and evening/used in morning or evening/not used in morning and evening) were considered as potential predictors. For a rolling window of 7 days, we also calculated the PEF average, standard deviation, maximum and minimum and added these as predictors. This rolling window consisted of the current day and all 6 preceding days. The PEF personal best was determined per patient during a run-in period of 4 weeks and added to the models. Lastly, we constructed and added first differences (the difference in today’s measurement with respect to yesterday’s measurement) and lags (yesterday’s measurement) for PEF, nocturnal awakening, and use of $\upbeta$2-reliever.

### Model development

Demographics and descriptive statistics of predictors (i.e., age, sex, mean PEF, PEF % personal best, nocturnal awakening, and use of $\upbeta$2-reliever) were calculated for each individual patient over their respective observational periods.

Missing values were interpolated based on previous and succeeding values and the data was normalized. The first ML model developed through supervised learning was a gradient boosted decision trees (XGBoost) model. This model was chosen as it is one of the most popular ML techniques, and it performs well for a wide selection of problems, including time series prediction^33^. The XGBoost model estimates many decision-trees sequentially. This is also called boosting. These decision tree predictions are combined into an ensemble model to arrive at the final predictions. The sequential training makes the XGBoost model faster and more efficient than other tree-based algorithms, such as random forest. A downside of this model is that, due to its complexity, it becomes hard to interpret. Moreover, when the missingness is high, tuning an XGBoost model may become increasingly difficult, which is less of an issue with other tree-based models like random forest.

Second, we trained an outlier detection model (one class SVM with Radial Basis Kernel)^34^. The one class SVM aims to find a frontier that delimits the contours of the original distribution. By estimating this frontier, it can identify whether a new data point falls outside of the original distribution and should therefore be classified as ‘irregular’. An advantage of this model is that it is particularly apt at dealing with the low event rate in the asthma data. A downside of this model is that it does not provide probability estimates like a regular support vector machine and we therefore must base its predictive performance on its classification metrics only (see below).

Additionally, we developed a prediction model using logistic regression as the popular classical prediction counterpart of these two ML models. Logistic regression assumes a probability distribution for the outcome variable and models the log-odds of each patient experiencing the outcome linearly. The log-odds are converted into probabilities via the logistic function. Logistic regression is an inherently interpretable technique and a hallmark of classical prediction modelling^35,36^. Due to its linearity restriction, it may however not provide the level of complexity needed to adequately model certain prediction problems. Machine learning methods, like XGBoost and one class SVM, provide more flexibility, which comes at a cost of the interpretability of these methods.

The hyperparameters of the XGBoost, one class SVM, and logistic regression models (see additional Table A4) were set using a full grid search and 5 × 5-fold cross-validation (stratified by patient) on the development cohort. We trained the final models using all data with optimized hyperparameters. We compared these model outcomes with a clinical rule that is currently proposed as action point in an asthma action plan by the British Thoracic Society: start oral corticosteroids treatment if PEF < 60% of personal best^2,5^.

### Model performance

After completing model development on the development cohort, all models and the clinical rule were applied to the validation cohort. The discriminative performance of the models producing probabilities (XGBoost and logistic regression) was measured via the area under the receiver operating characteristic curve (AUC) and histograms of the probability distributions were plotted. We applied the DeLong test to compare the AUCs from these two models. Calibration was assessed graphically and quantified through the calibration slope and intercept^26^. Confidence intervals were obtained through bootstrapping (based on a 1000 iterations). Sensitivity, specificity, positive predictive value (PPV), and negative predictive value (NPV) were calculated for all models at the following probability thresholds (the cut-off point at which probabilities are converted into binary outcomes): 0.1% and 0.2%. These were chosen as they circle the prevalence rate of the outcome in our data. For a fair comparison with the clinical rule, we also calculated these performance metrics (sensitivity, specificity, etc.) for the XGBoost and logistic regression models at the probability thresholds producing the same number of positive predictions as produced by the one class SVM and the clinical rule.

### Sensitivity analysis

We performed a sensitivity analysis for predicting exacerbations within 4 and 8 days as opposed to 2 days (Table 4). This enabled us to study the effect of a variation in the length of the outcome window on the models’ discrimination and calibration capacities.

Second, we performed a sensitivity analysis to assess the effect of the number of lags on model performance. For this analysis, we varied the number of lags from 1 to 5 for the models predicting exacerbations within 2 days. For the XGBoost and logistic regression model, the AUC was compared. For the one class SVM model, the sensitivity, specificity, PPV, and NPV were compared.

### Software

All analyses were performed in Python 3.8.0. with R 3.6.3 plug-ins to obtain calibration results. The key functions and libraries can be found in additional file 2. The complete code is available on request.

### Ethics approval and consent to participate

Ethics approval was obtained for the original data collection. These studies were conducted in accordance with the principles of the Declaration of Helsinki on biomedical research. The protocols were approved by the Otago and Canterbury ethics committees and all patients gave written informed consent prior to participation.

## Supplementary Information

Supplementary Information.

## Data Availability

The datasets analyzed during the current study are not publicly available due to privacy restrictions, but are available to reviewers on reasonable request.
