# Supplementary material for: Machine learning did not beat logistic regression in time series prediction for severe asthma exacerbations
Source: Sci Rep. 2022 Nov 27;12:20363. doi: 10.1038/s41598-022-24909-9 (PMC9701686; doi:10.1038/s41598-022-24909-9)
Supplement: Supplementary file 1 — Supplementary Information. [file 41598_2022_24909_MOESM1_ESM.docx]

ADDITIONAL TABLES

| **Table A1. Hyper parameters machine learning and logistic regression models** | | | | |
| --- | --- | --- | --- | --- |
| Hyperparameters | Values | Parameters for exacerbations within 2 days | Parameters for exacerbations within 4 days | Parameters for exacerbations within 8 days |
| XGBoost |  |  |  |  |
| Number of trees | {25, 100, 200} | 25 | 25 | 25 |
| Maximum depth | {1, 3, 5, 7, 8, 9} | 3 | 3 | 3 |
| Learning rate | {0.1, 0.3, 0.5, 0.7, 0.9} | 0.7 | 0.9 | 0.7 |
| One class SVM |  |  |  |  |
| nu | {0.001, 0.0015, 0.002, 0.004, 0.006, 0.008, 0.01} | 0.001 | 0.001 | 0.001 |
| gamma | {0.001, 0.01, 0.1, 1} | 0.001 | 0.001 | 0.001 |
| Logistic regression |  |  |  |  |
| Penalty | {0.1, 0.2, 0.3, 0.4, 0.5, 0.6, 0.7, 0.8, 0.9} | 0.9 | 0.7 | 0.9 |
|  | | | | |

| **Table A2. Discrimination and calibration for predicting exacerbation within 4 days** | | | |
| --- | --- | --- | --- |
|  | AUC | Calibration intercept | Calibration slope |
| XGBoost | 0.77 (0.74, 0.79) | 0.57 (0.45, 0.68) | 0.48 (0.43, 0.52) |
| Logistic regression | 0.85 (0.83, 0.86) | 0.65 (0.54, 0.75) | 1.07 (1.0, 1.14) |
| Abbreviations: *XGBoost* gradient boosted decision trees, *AUC* Area Under the Receiver Operating Characteristics Curve | | | |

| **Table A3. Threshold specific performance metrics for predicting exacerbation within 4 days** | | | | | |
| --- | --- | --- | --- | --- | --- |
| Probability threshold | Model | Sensitivity | Specificity | PPV | NPV |
| 0.001 | XGBoost | 0.77 (285/372) | 0.6 (23638/39720) | 0.02 (285/16367) | 1.0 (23638/23725) |
|  | Logistic regression | 0.99 (370/372) | 0.06 (2564/39720) | 0.01 (370/37526) | 1.0  (2564/2566) |
| 0.002 | XGBoost | 0.69 (258/372) | 0.76 (30234/39720) | 0.03 (258/9744) | 1.0 (30234/30348) |
|  | Logistic regression | 0.96 (356/372) | 0.36 (14385/39720) | 0.01 (356/25691) | 1.0 (14385/14401) |
| Resulting in 5217 positive predictions^**^ | One class SVM | 0.3 (113/372) | 0.87 (34616/39720) | 0.02 (113/5217) | 0.99 (34616/34875) |
|  | XGBoost | 0.58 (215/372) | 0.87 (34543/39720) | 0.04 (215/5392) | 1.0 (34543/34700) |
|  | Logistic regression | 0.62 (229/372) | 0.88 (34810/39720) | 0.04 (229/5139) | 1.0 (34810/34953) |
| Resulting in 138 positive predictions^**^ | Clinical rule^*^ | 0.03 (13/372) | 1.0 (39595/39720) | 0.09  (13/138) | 0.99 (39595/39954) |
|  | XGBoost | 0.03 (11/372) | 1.0 (39593/39720) | 0.08  (11/138) | 0.99 (39593/39954) |
|  | Logistic regression | 0.06 (22/372) | 1.0 (39604/39720) | 0.16  (22/138) | 0.99 (39604/39954) |
| *Peak Expiratory Flow < 60% personal best  **This threshold is set so that the XGBoost and logistic regression models produce the same number of positive predictions as the one class SVM or clinical rule.  Abbreviations: *SVM* Support Vector Machine, *XGBoost* gradient boosted decision trees, *PPV* Positive Predictive Value, *NPV* Negative Predictive Value | | | | | |

| **Table A4. Discrimination and calibration for predicting exacerbation within 8 days** | | | |
| --- | --- | --- | --- |
|  | AUC | Calibration intercept | Calibration slope |
| XGBoost | 0.7 (0.68, 0.72) | 0.58 (0.5, 0.66) | 0.43 (0.39, 0.47) |
| Logistic regression | 0.81 (0.79, 0.82) | 0.59 (0.52, 0.67) | 1.11 (1.04, 1.17) |
| Abbreviations: *XGBoost* gradient boosted decision trees, *AUC* Area Under the Receiver Operating Characteristics Curve | | | |

| **Table A5. Threshold specific performance metrics for predicting exacerbation within 8 days** | | | | | |
| --- | --- | --- | --- | --- | --- |
| Probability threshold | Model | Sensitivity | Specificity | PPV | NPV |
| 0.001 | XGBoost | 0.86 (615/712) | 0.25 (9817/39380) | 0.02 (615/30178) | 0.99  (9817/9914) |
|  | Logistic regression | 1.0 (712/712) | 0.0  (149/39380) | 0.02 (712/39943) | 1.0  (149/149) |
| 0.002 | XGBoost | 0.79 (563/712) | 0.37 (14592/39380) | 0.02 (563/25351) | 0.99 (14592/14741) |
|  | Logistic regression | 1.0 (711/712) | 0.03 (1224/39380) | 0.02 (711/38867) | 1.0  (1224/1225) |
| Resulting in 5217 positive predictions^**^ | One class SVM | 0.3 (211/712) | 0.87 (34374/39380) | 0.04  (211/5217) | 0.99 (34374/34875) |
|  | XGBoost | 0.41 (293/712) | 0.87 (34385/39380) | 0.06  (293/5288) | 0.99 (34385/34804) |
|  | Logistic regression | 0.5 (357/712) | 0.88 (34512/39380) | 0.07  (357/5225) | 0.99 (34512/34867) |
| Resulting in 138 positive predictions^**^ | Clinical rule^*^ | 0.03 (21/712) | 1.0 (39263/39380) | 0.15  (21/138) | 0.98 (39263/39954) |
|  | XGBoost | 0.02 (17/712) | 1.0 (39259/39380) | 0.12  (17/138) | 0.98 (39259/39954) |
|  | Logistic regression | 0.05 (35/712) | 1.0 (39277/39380) | 0.25  (35/138) | 0.98 (39277/39954) |
| *Peak Expiratory Flow < 60% personal best  **This threshold is set so that the XGBoost and logistic regression models produce the same number of positive predictions as the one class SVM or clinical rule.  Abbreviations: *SVM* Support Vector Machine, *XGBoost* gradient boosted decision trees, *PPV* Positive Predictive Value, *NPV* Negative Predictive Value | | | | | |

| **Table A6. Discrimination for predicting exacerbation within 2 days with varying number of lags** | | |
| --- | --- | --- |
|  | AUC XGBoost | AUC Logistic regression |
| 1 lag | 0.81 (0.78, 0.84) | 0.88 (0.86, 0.90) |
| 2 lags | 0.81 (0.78, 0.84) | 0.88 (0.86, 0.90) |
| 3 lags | 0.82 (0.79, 0.85) | 0.88 (0.86, 0.90) |
| 4 lags | 0.82 (0.80, 0.85) | 0.88 (0.85, 0.90) |
| 5 lags | 0.85 (0.82, 0.87) | 0.88 (0.85, 0.90) |
| Abbreviations: *XGBoost* gradient boosted decision trees, *AUC* Area Under the Receiver Operating Characteristics Curve | | |

| **Table A7. Classification of one class SVM for predicting exacerbation within 2 days with varying number of lags** | | | | |
| --- | --- | --- | --- | --- |
|  | Sensitivity | Specificity | PPV | NPV |
| 1 lag | 0.34 (64/188) | 0.87 (34751/39904) | 0.01 (64/5217) | 1.0 (34751/34875) |
| 2 lags | 0.45 (85/188) | 0.85 (33985/39904) | 0.01 (85/6004) | 1.0 (33985/34088) |
| 3 lags | 0.46 (86/188) | 0.83 (33296/39904) | 0.01 (86/6694) | 1.0 (33296/33398) |
| 4 lags | 1.0 (188/188) | 0.02 (840/39904) | 0.0 (188/39252) | 1.0 (840/840) |
| 5 lags | 0.99 (186/188) | 0.03 (1022/39904) | 0.0 (186/39068) | 1.0 (1022/1024) |
| Abbreviations: *SVM* Support Vector Machine, *PPV* Positive Predictive Value, *NPV* Negative Predictive Value | | | | |

ADDITIONAL FIGURES

| 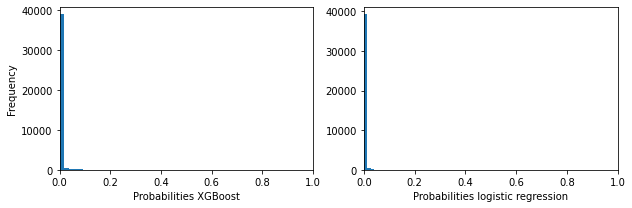 |
| --- |
| **Figure A1. Histogram of probability predictions for a) XGBoost model and b) logistic regression model.** |

| a)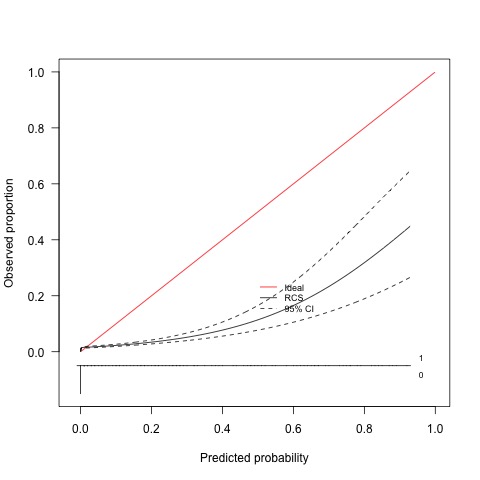 | b)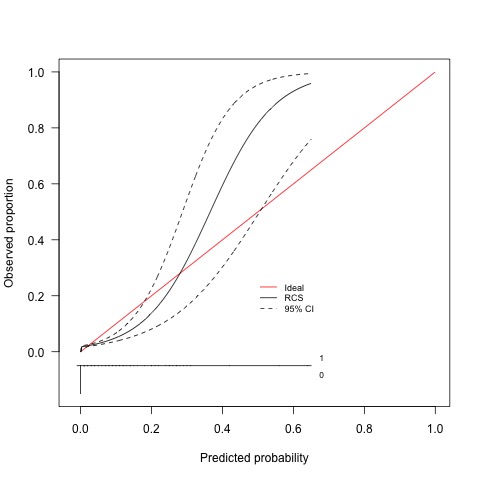 |
| --- | --- |
| **Figure A2. Calibration curves for a) XGBoost model and b) logistic regression model.** | |

CODE

Employed python packages for model training

# LOAD PACKAGES

import pandas as pd

import matplotlib.pyplot as plt

import numpy as np

from sklearn.model_selection import train_test_split

from sklearn import svm

from sklearn.linear_model import LogisticRegression

from xgboost import XGBClassifier

Calculating predictions expert rule in python

#######################################################################

# Expert rule

#######################################################################

test_pred_expert = (test_X.pefr < 0.6*test_X.max_pefr)*1

Training model and calculating (probability) predictions logistic regression in R

#######################################################################

# Logistic regression

#######################################################################

model = LogisticRegression(random_state=0, C=1, max_iter=10000)

best_score_logit, best_params_logit = optimize_hyperpar(param_grid={'C':[0.1,0.2,0.3,0.4,0.5,0.6,0.7,0.8,0.9]}, X=train_X, y=train_y, k_folds=5, model=model, calibrated='', cont=cont_cols, cat=cat_cols)

# train model

model.set_params(**best_params_logit)

model.fit(train_X.drop('subject',axis=1), train_y)

# test model

test_pred_logit = model.predict(test_X.drop('subject',axis=1))

test_prob_logit = model.predict_proba(test_X.drop('subject',axis=1))

Training model and calculating predictions one class SVM in python

#######################################################################

# one class SVM

#######################################################################

model = svm.OneClassSVM(nu=nu)

best_score_one_class_SVM, best_params_one_class_SVM = optimize_hyperpar(param_grid={'nu':[0.001,0.0015,0.002,0.004,0.006,0.008,0.01],'gamma':[0.001, 0.01, 0.1]},

X=train_X, y=train_y, k_folds=5, model=model, calibrated='', cont=cont_cols, cat=cat_cols)

print(best_params_one_class_SVM, best_score_one_class_SVM)

# train model

model.set_params(**best_params_one_class_SVM)

model.fit(train_X.drop('subject',axis=1), train_y)

# test model

test_pred_onesvm = model.predict(test_X.drop('subject',axis=1))

test_pred_onesvm[test_pred_onesvm==1] = 0

test_pred_onesvm[test_pred_onesvm==-1] = 1

Training model and calculating (probability) predictions XGBoost in python

#######################################################################

# XGBoost

#######################################################################

model = XGBClassifier(random_state=42)

best_score_XGBoost, best_params_XGBoost = optimize_hyperpar(param_grid={'n_estimators':[25,50,100], 'learning_rate':[0.1,0.3,0.5,0.7,0.9], 'max_depth':range(1,11,2)}, X=train_X, y=train_y, k_folds=5, model=model, cont=cont_cols, cat=cat_cols)

# train model

model.set_params(**best_params_XGBoost)

model.fit(train_X.drop('subject',axis=1), train_y)

# test model

test_pred_XGBoost = model.predict(test_X.drop('subject',axis=1))

test_prob_XGBoost = model.predict_proba(test_X.drop('subject',axis=1))

Employed python packages for model output

import pandas as pd

import matplotlib.pyplot as plt

import numpy as np

from sklearn.metrics import roc_curve

from sklearn.metrics import precision_recall_curve

from sklearn.metrics import auc

import statsmodels.api as sm

import statsmodels.formula.api as smf

import rpy2.robjects as robjects

from rpy2.robjects import numpy2ri, pandas2ri

numpy2ri.activate()

pandas2ri.activate()

Python function for running calibration plot in R

def calibration_stats(y, prob_y, model):

rstring = """

function(y, prob_y, model){

library(rms)

source('CalibrationCurves/val.prob.ci.2.R')

source('CalibrationCurves/ci.auc.R')

source('CalibrationCurves/auc.nonpara.mw.R')

jpeg(file=paste('../plots/calibration_',model,'.jpeg',sep=''))

val.prob.ci.2(prob_y, y, smooth='rcs', pl=TRUE)

dev.off()

}

"""

cali_plot = robjects.r(rstring)

output = cali_plot(y, prob_y, model)

return(output)

Python function for producing bootstrapped AUC confidence interval

def bootstrap_AUC(n, y, prob_y, curve='ROC'):

y.index = range(0,len(y))

prob_y.index = range(0,len(y))

n_bootstraps = n

rng_seed = 42 # control reproducibility

rng = np.random.RandomState(rng_seed)

bootstrapped_scores = []

for i in range(n_bootstraps):

# bootstrap by sampling with replacement on the prediction indices

indices = rng.randint(0, prob_y.shape[0], prob_y.shape[0])

if len(np.unique(y[indices])) < 2:

# We need at least one positive and one negative sample for ROC AUC

# to be defined: reject the sample

continue

if curve=='ROC':

x_axis, y_axis, thresholds = roc_curve(y[indices], prob_y[indices])

elif curve=='PRC':

y_axis, x_axis, thresholds = precision_recall_curve(y[indices], prob_y[indices])

score = auc(x_axis, y_axis)

bootstrapped_scores.append(score)

sorted_scores = np.array(bootstrapped_scores)

sorted_scores.sort()

# Computing the lower and upper bound of the 90% confidence interval

# You can change the bounds percentiles to 0.025 and 0.975 to get

# a 95% confidence interval instead.

confidence_lower = sorted_scores[int(0.05 * len(sorted_scores))]

confidence_upper = sorted_scores[int(0.95 * len(sorted_scores))]

return(confidence_lower, confidence_upper)

Python function for producing discrimination and calibration statistics

def performance_table(probabilities, models):

table = pd.DataFrame(columns=['AUROC','AUPRC','calibration int', 'calibration slope'])

for m in models:

p = probabilities.loc[:,m]

y = probabilities.label

# discrimination

precision, recall, _ = precision_recall_curve(y, p)

fpr, tpr, _ = roc_curve(y, p)

auroc_CI = bootstrap_AUC(1000, y, p, curve='ROC')

auprc_CI = bootstrap_AUC(1000, y, p, curve='PRC')

# calibration

logit = np.log(p/(1-p))

df = pd.DataFrame(np.transpose([y,logit]),columns=['y','logit'])

mod_slope = smf.glm('y~logit', data=df, family=sm.families.Binomial()).fit()

mod_interc = smf.glm('y~1', data=df, offset=logit, family=sm.families.Binomial()).fit()

#form table

table = table.append({

'AUROC': str(round(auc(fpr, tpr),2)) + ' ' + str(tuple(round(i,2) for i in auroc_CI)),

'AUPRC': str(round(auc(recall, precision),2)) + ' ' + str(tuple(round(i,2) for i in auprc_CI)),

'calibration int': str(round(mod_interc.params[0],2)) + ' ' + str(tuple(round(i,2) for i in list(np.array(mod_interc.conf_int(alpha=0.05))[0,:]))),

'calibration slope': str(round(mod_slope.params[1],2)) + ' ' + str(tuple(round(i,2) for i in list(np.array(mod_slope.conf_int(alpha=0.05))[1,:])))

},ignore_index=True)

table.index = models

return table

Python function for threshold reliant metrics (sensitivity, specificity, etc.)

def performance_table_thresholds(probabilities, models, thresholds):

true_y = probabilities.label

columns = ['sensitivity','specificity','PPV','NPV']

index = [m + ' ' + str(t) for t in thresholds for m in models]

table = pd.DataFrame(columns=columns, index=index)

for m in models:

for t in thresholds:

pred_y = np.array(probabilities.loc[:,m]>t)

table.loc[m+' '+str(t),'sensitivity'] = str(np.round(compute_sensitivity(true_y,pred_y),2)) + ' (' + str((true_y[pred_y==1]==1).sum()) + '/' + str((true_y==1).sum()) + ')'

table.loc[m+' '+str(t),'specificity'] = str(np.round(compute_specificity(true_y,pred_y),2)) + ' (' + str((true_y[pred_y==0]==0).sum()) + '/' + str((true_y==0).sum()) + ')'

table.loc[m+' '+str(t),'PPV'] = str(np.round(compute_PPV(true_y,pred_y),2)) + ' (' + str((true_y[pred_y==1]==1).sum()) + '/' + str((pred_y==1).sum()) + ')'

table.loc[m+' '+str(t),'NPV'] = str(np.round(compute_NPV(true_y,pred_y),2)) + ' (' + str((true_y[pred_y==0]==0).sum()) + '/' + str((pred_y==0).sum()) + ')'

return table

Python function for threshold reliant metrics at a given number of positive predictions

def performance_table_fixed_positives(probabilities, models, ref):

true_y = probabilities.label

columns = ['sensitivity','specificity','PPV','NPV']

index = [m + ' ' for m in models]

table = pd.DataFrame(columns=columns, index=index)

# find threshold

tot_pos = probabilities.loc[:,ref].sum()

print('positive predictions is:',tot_pos)

for m in models:

if len(probabilities.loc[:,m].value_counts())>2:

pos_min, start, stop, step = [1,0,1,0.1]

while pos_min>0:

pos_array = np.array([(probabilities.loc[:,m]>i).sum() for i in np.arange(start,stop,step=step)])

pos_min = min(np.abs(pos_array-tot_pos))

t = np.arange(start,stop,step=step)[np.abs(pos_array-tot_pos)==pos_min]

if len(t)==1:

start = t-step

stop = t+step

step = step/10

if len(t)>1: t = t[0]

else: t = t[0]

print('threshold for ',m,'is:',t)

pred_y = np.array(probabilities.loc[:,m]>t)

else:

pred_y = probabilities.loc[:,m]

table.loc[m+' ','sensitivity'] = str(np.round(compute_sensitivity(true_y,pred_y),2)) + ' (' + str((true_y[pred_y==1]==1).sum()) + '/' + str((true_y==1).sum()) + ')'

table.loc[m+' ','specificity'] = str(np.round(compute_specificity(true_y,pred_y),2)) + ' (' + str((true_y[pred_y==0]==0).sum()) + '/' + str((true_y==0).sum()) + ')'

table.loc[m+' ','PPV'] = str(np.round(compute_PPV(true_y,pred_y),2)) + ' (' + str((true_y[pred_y==1]==1).sum()) + '/' + str((pred_y==1).sum()) + ')'

table.loc[m+' ','NPV'] = str(np.round(compute_NPV(true_y,pred_y),2)) + ' (' + str((true_y[pred_y==0]==0).sum()) + '/' + str((pred_y==0).sum()) + ')'

return table
